# Supplementary material for: Tunable colloidal swarmalators with hydrodynamic coupling
Source: Nat Commun. 2025 Dec 8;16:10984. doi: 10.1038/s41467-025-66830-5 (PMC12690114; doi:10.1038/s41467-025-66830-5)
Supplement: Supplementary file 1 — Supplementary Information [file 41467_2025_66830_MOESM1_ESM.pdf]

Supplementary Information to Tunable colloidal swarmalators with hydrodynamic coupling

Veit-Lorenz Heuthe[1,*], Priyanka Iyer[2,*], Gerhard Gompper[2], and Clemens Bechinger[1,†]

[1]Fachbereich Physik, Universität Konstanz, D-78465 Konstanz, Germany

[2]Theoretical Physics of Living Matter, Institute for Advanced Simulation, Forschungszentrum Jülich, D-52425 Jülich, Germany

[*]These authors contributed equally.

[†]Corresponding author. Email: clemens.bechinger@uni-konstanz.de

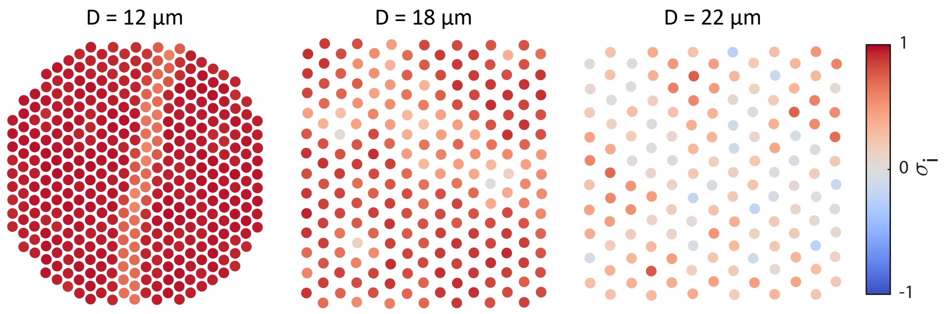

Fig. S1: Distance dependence of synchronization. Simulation snapshots of oscillators with their reference points arranged on a lattice for different lattice spacing D. The oscillators are colored according to their synchronization with neighbors σ_i.

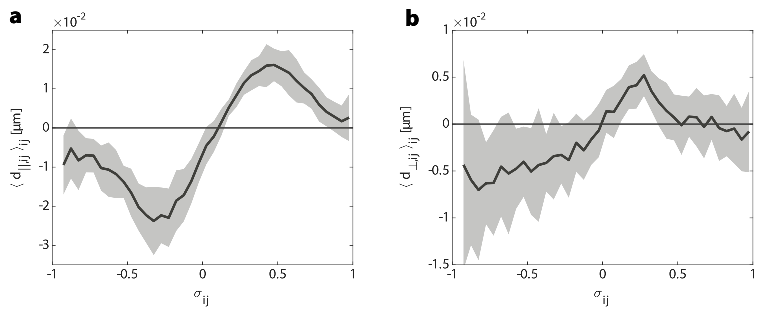

Fig. S2: Displacement as a function of synchronization. **a** $d_{\parallel,ij}$ and **b** $d_{\perp,ij}$ as a function of the synchronization σ_{ij} between neighbors averaged over reference point distances $|\mathbf{q}_{ij}|$ between 8 - 15 µm. The gray shaded areas show the standard deviation over the distance $|\mathbf{q}_{ij}|$. Source data are provided as a Source Data file.

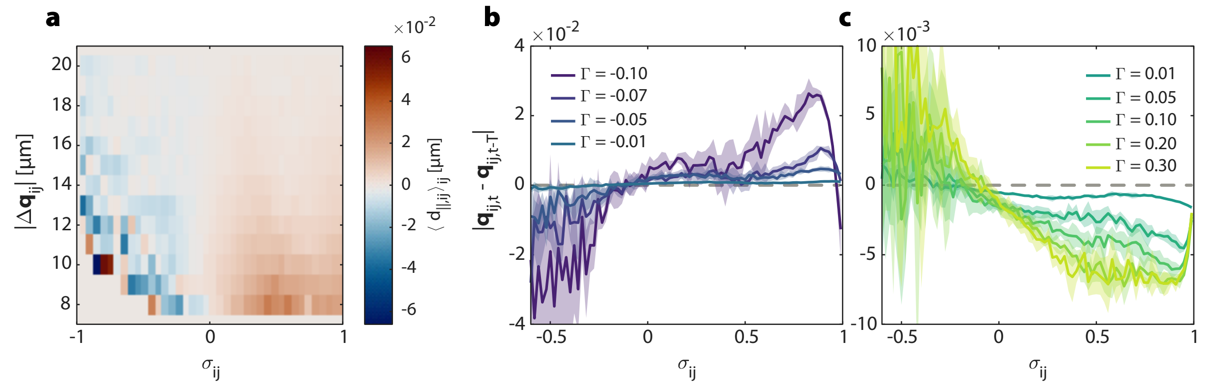

Fig. S3: Synchronization-dependent motion in simulations. **a** Heat map of the mean parallel displacement $\langle d_{\|,ij} \rangle_{\|,ij}$ for different synchronizations and distances of Delaunay neighbors in simulations. **b, c** Relative velocity of Delaunay neighbors in simulations for different values of Γ (color of the curve). For $\Gamma < 0$, synchronized swarmalators show repulsive interactions, while $\Gamma > 0$, synchronized swarmalators attract each other. The data shows very good qualitative agreement with the experimental data in the main text (Fig. 3e,f). Source data are provided as a Source Data file.

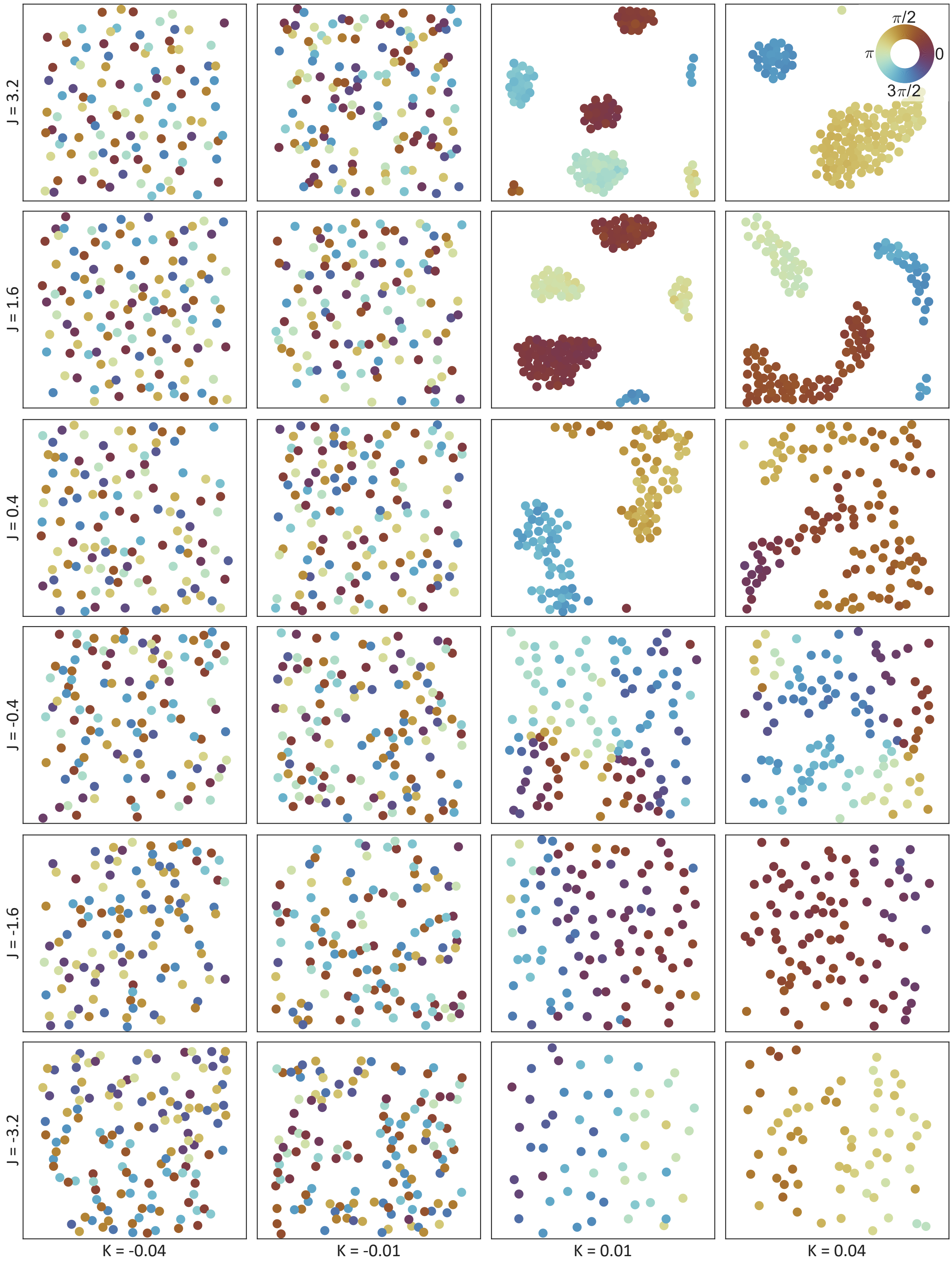

Fig. S4: Full phase diagram of the swarmalator model. K and J are the parameters governing the distance-dependent synchronization and synchronization-dependent attraction or repulsion, respectively (see equations (3) and (4)). The colors denote the phases of the swarmalators as indicated in the top right corner.

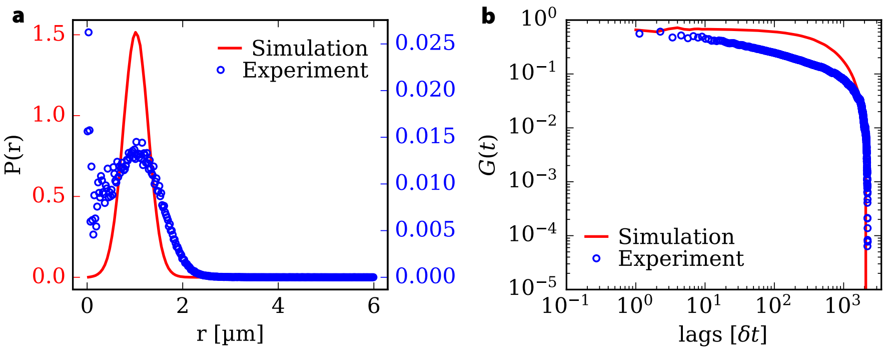

Fig. S5: Single-particle dynamics. a Distribution of the distance between the swarmalator and its reference position in simulations and experiments. The ABP oscillates around the reference point at an average distance of approximately 1 µm. **b** Particle angular velocity auto-correlation in simulations and experiments show very good agreement when the simulation uses the same update scheme (for the laser) as is in the experiment. Here, in both simulation and experiment, passive diffusion coefficient $D_t \simeq 0.03\mu\text{m/s}^2$,$\delta t = 0.4\text{s}$ and particle speed $u_0 = 2.5\mu\text{m/s}$. Source data are provided as a Source Data file.

1. Single particle motion

Here, we show that the update of the orientation vector $\mathbf{e_i}(t)$ based on the laser position $\mathbf{l_i}(t)$, i.e. $\boldsymbol{e}_i(t) = (\mathbf{r}_i(t) - \mathbf{l_i}(t))/|\mathbf{r}_i(t) - \mathbf{l_i}(t)|$, gives the right dynamics for the single particle motion. In Fig. S5, we compare single-particle motion in simulation and experiment via the angular-velocity auto-correlation $G(t) = \langle\dot{\theta}(t)\dot{\theta}(0)\rangle/\langle\dot{\theta}(0)^2\rangle$, which gives a good agreement and thereby validates the implementation of the update scheme in the simulation.

2. Probing two-particle interactions

To develop the simulation model, a series of additional experiments were performed to determine the simulation parameters which best match the experimental system. Here, an active particle and a passive particle were placed at a initial separation of $\sim$ 30 µm and then the active particle was driven in the direction of the passive particle. This was repeated for different active particle velocities, and the velocity of the passive particle was measured to probe the strength of the hydrodynamic interactions between the particles. This experiment was then replicated in simulations using identical initial conditions. It was observed that a model with only squirmer interactions significantly underestimates the hydrodynamic forces, as shown in Fig. S6 (black lines) due to the fast decay of the (far-field) hydrodynamic forces $\sim r^{-3}$. To address this issue, lubrication forces were incorporated into the simulation model. The lubrication force added described by the flow field

$$\mathbf{U}_{lub,j}(r) = -\frac{a}{h}(\dot{\mathbf{r}} \cdot \hat{\mathbf{r}})\Theta(r)\hat{\mathbf{r}} \tag{1}$$

where $\dot{\mathbf{r}} = \dot{\mathbf{r}}_i - \dot{\mathbf{r}}_j$ is the relative velocity between particle j and i, $h = r - 2a$ is the shortest distance between the surface of the two particles. A cutoff function of the form $\Theta(r) = \exp(-r/R_0)$ captures the finite range of the force, where different the parameter R_0 is varied to match the simulation to the experimental data. For the two-particle experiment, we find that a value $R_0 \simeq 2.0a$ matches the experimentally observed long decay of the hydrodynamic forces (blue lines Fig. S6). However, the grid experiments (see Fig. 1 of main text), suggest a much slower decay of the hydrodynamic forces. Thus, we ultimately selected a value of $R_0 \sim 3.5a$ (Fig. S6 green lines) to achieve reasonable agreement between the simulation data for both the two-particle interaction and the grid experiments. Given the simplicity of the model, our goal is primarily a qualitative agreement with experimental observations, as a quantitative match is challenging at this level of description.

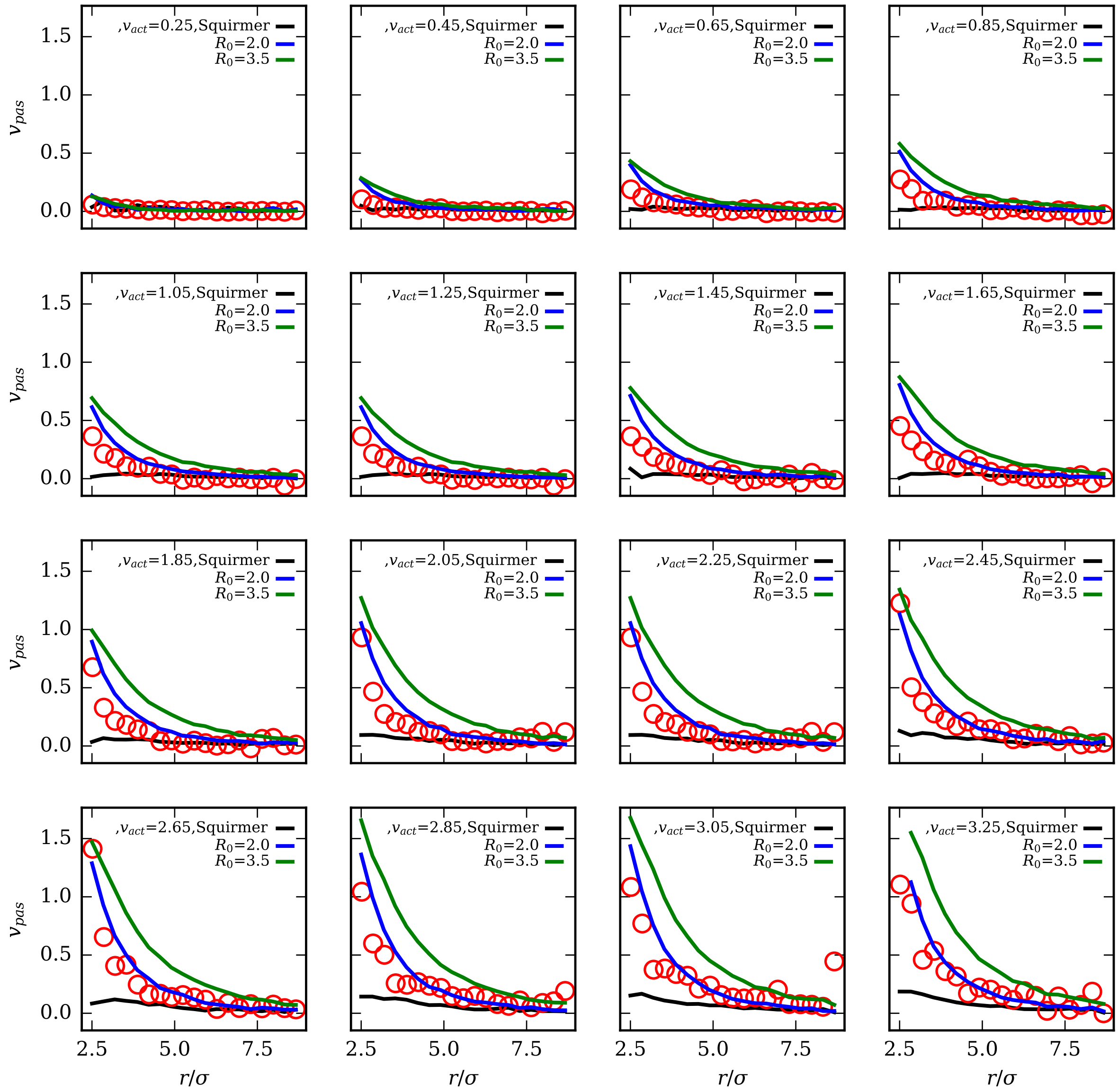

Fig. S6: Binary interactions between an active particle and a passive particle. Velocity v_{pas} of the passive particle as a function of the separation r from the active particle. The different sub-figures correspond to different velocities of the driven particle. Experimental data is shown with red circles, results from the simulation data by solid lines, with different choices of the decay range R_0 (in units of particle radius a). Source data are provided as a Source Data file.

3. Synchronization domains

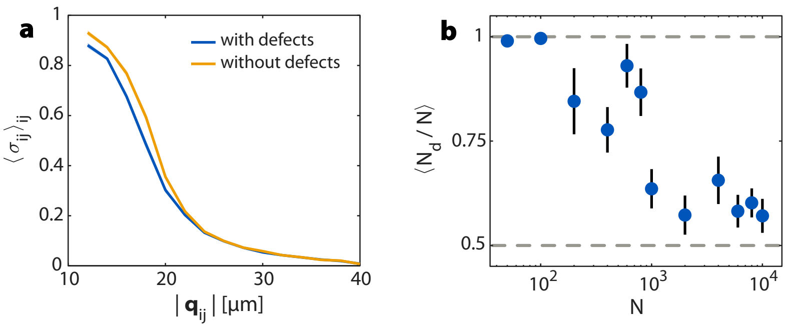

Fig. S7: Synchronization organizes in domains. **a** Mean synchronization vs the distance of reference positions $|\mathbf{q}_{ij}|$ on a grid with defects (blue, as in Fig. 1g in the main text) and without defects (orange). **b** The mean synchronization domain size (with standard deviation) normalized by the the system size $\langle N_D/N \rangle$ over the total number of particles in the system N. Source data are provided as a Source Data file.

4. Hydrodynamic synchronization of oscillations

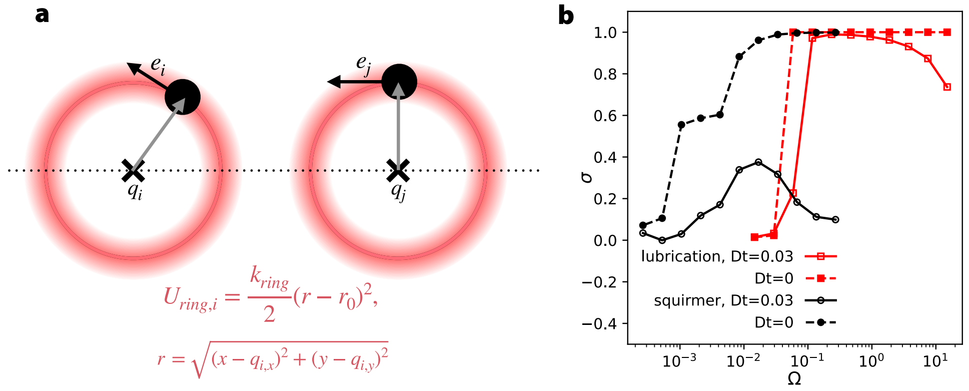

Fig. S8: **a** Schematic for the motion of two oscillators confined to move on a circle via a ring potential with strength k_{ring}. **b** Mean synchronization of an oscillator interacting through lubrication and squirmer forces, shown with noise (solid lines) and without noise (dashed lines). Here $\Omega = F_{\mathrm{HI}}/k_{ring}r_0$ measures the deviation from the circle radius r_0, where $F_{\mathrm{HI}} = s\gamma/R^3$ and $F_{\mathrm{HI}} = au_0\gamma/(R-2a)$ for squirmer and lubrication forces respectively. In the absence of noise, synchronization decays monotonically to zero when the oscillators are unable to vary their radial position (i.e., for $\Omega < 0.05$). With noise, however, a maximum emerges, such that intermediate radial flexibility leads to the strongest synchronization. Because the squirmer interactions are weak, the system does not reach perfect synchronization for $D_t \neq 0$. Here, the distance between the reference positions is fixed at $R = 8$, with seven oscillators arranged on a hexagonal grid. Synchronization is computed for the central oscillator [equation (1) main text] and averaged over 5000 delay times. Source data are provided as a Source Data file.

To understand the synchronization of the oscillators, we consider a simplified model involving two swarmalators rotating around fixed reference positions. We assume that the reference points are fixed at $\mathbf{q}_i = (0,0)$ and $\mathbf{q}_j = (R,0)$, with the two swarmalators performing a circular trajectory around their respective reference points. The position of the swarmalators in polar coordinates are given by $\mathbf{r}_i(t) = r(\cos\theta, \sin\theta)$ and $\mathbf{r}_j(t) = (R + r\cos(\theta+\epsilon), r\sin(\theta+\epsilon))$, where ϵ is the phase difference between the two swarmalators. Their orientation vectors are then given by $\mathbf{e}_i(t) = (-\sin\theta, \cos\theta)$ and $\mathbf{e}_j(t) = (-\sin(\theta+\epsilon), \cos(\theta+\epsilon))$, see Fig. S8(a). We now consider the influence of particle j on i, assuming their trajectories are perfectly circular, i.e. $\dot{r} = 0, r = r_0$. In this limit, the equation of motion of particle i (in the overdamped limit) can be approximated as

$$r_0\dot{\theta}_i \simeq (u_0\mathbf{e}_i + \mathbf{U}_{flow,j} + \mathbf{U}_{lub,j} + \sqrt{2D_t}\boldsymbol{\zeta}_i)\cdot\mathbf{e}_i \quad (2)$$

Simplifying and retaining leading order terms in the limit $|\epsilon| \ll 1$ gives

$$\dot{\theta}_i \simeq \omega_0 + \frac{s}{2R^3 r_0}[\cos\epsilon - 3\cos 2\theta + 6\epsilon\cos\theta\sin\theta] + \frac{au_0}{(R-2a)r_0}\epsilon\cos\theta\sin\theta\ \Theta(R) + \sqrt{2D_t}\boldsymbol{\zeta}_i\cdot\mathbf{e}_i \tag{3}$$

Similarly, for θ_j,

$$\dot{\theta}_j \simeq \omega_0 + \frac{s}{2R^3 r_0}[\cos\epsilon - 3\cos 2\theta + 6\epsilon\cos\theta\sin\theta] - \frac{au_0}{(R-2a)r_0}\epsilon\cos\theta\sin\theta\ \Theta(R) + \sqrt{2D_t}\boldsymbol{\zeta}_i\cdot\mathbf{e}_i \tag{4}$$

Two important observations can be made. First, the interactions due to squirmer forces ($1/R^3$ term) are symmetric under the exchange of i and j, and therefore cannot lead to synchronization in this model [1]. Second, while lubrication forces are not symmetric under exchange of i and j, their average over one rotation vanishes, which also implies the absence of synchronization. Nevertheless, in our simulations we observe strong synchronization. We conjecture that additional degrees of freedom—in particular, radial motion—is necessary to induce synchronization [2, 3].

To test this idea, we consider a simplified model of oscillators interacting through lubrication and/or squirmer forces, constrained to move along nearly circular trajectories by a harmonic (ring-shaped) potential of strength k_{ring} [Fig. S8(a)], which we vary systematically. For simplicity, we implement a perfect update scheme: the orientation vector $\mathbf{e}_i$ for the swarmalator i is always perpendicular to $\mathbf{r}_i - \mathbf{q}_i$, as assumed in the calculations above. We then define the dimensionless parameter $\Omega = F_{\mathrm{HI}}/k_{ring}r_0$, where $F_{\mathrm{HI}} = s\gamma/R^3$ and $F_{\mathrm{HI}} = au_0\gamma/(R-2a)$ for squirmer and lubrication forces, respectively. The parameter Ω characterizes the strength of fluctuations (in units of r_0) around the circular orbit of radius r_0. As shown in Fig. S8(b), for small Ω (corresponding to nearly perfect circular trajectories), the system does not synchronize, consistent with Eqs. (3)–(4). In contrast, as Ω increases (with decreasing k_{ring}) to $\Omega \simeq 0.05$, the particles are able to adjust their radial positions sufficiently, leading to strong synchronization. When noise is present ($D_t \neq 0$, solid lines), a maximum in synchronization is also observed. Thus, the ability to adjust the radial position — is crucial for the oscillators to synchronize. Note that short-range synchronization is governed by lubrication forces, which generate strong synchronization even in the presence of noise, whereas the long-range behavior is determined by the squirmer interaction with its characteristic $1/R^3$ decay.

Given that the system can synchronize when radial degrees of freedom are allowed, we now use Eqs. (3) and (4) to motivate the phenomenological swarmalator model. The long-range behavior decays as $1/R^3$, whereas the short-range behavior exhibits a more complex dependence as we implement an ad-hoc cutoff function $\Theta(R)$ to match the experimental data. For simplicity, we then assume that the weakest coupling term is the long-range $1/R^3$ interaction, with a coupling strength K that depends on the magnitude of the underlying hydrodynamic interactions, such as the squirmer source-dipole strength s and lubrication forces. We then can use the general form [1]

$$\dot{\theta}_i(t) = \omega_0 + \sum_{j\neq i}\frac{K}{|\mathbf{q}_{ij}(t)|^3}\sin[\Delta\theta_{ij}(t)] + \xi_{i,\theta}(t). \tag{5}$$

Note that here this interaction shares the same distance dependence of hydrodynamic interactions of pulled bead (force monopole) near a no-slip substrate. However, the long-range $1/R^3$ coupling in our phenomenological swarmalator model arises from the flow fields of force-free neutral squirmers, and is thus fundamentally different from the $1/R^3$ hydrodynamic interactions of a pulled bead near a no-slip boundary [4, 5].

Figure S7a shows the mean synchronization $\langle\sigma_{ij}\rangle$ against the distance between reference positions $|\mathbf{q}_{ij}|$ without defects in comparison to the data from Fig. 1g in the main text, which was computed from systems with defects. The inclusion of defects yields a slightly lower synchronization for small reference point distances. The lower synchronization at shorter distances observed in experiments can therefore be understood as arising from the presence of defects, such as unresponsive or missing particles, whereas the simulation model operates under ideal conditions, leading to higher synchronization.

As mentioned in section 2.1 in the main text, the oscillators form domains of high synchronization when their reference points are arranged on a grid. We analyzed the domain sizes for different system sizes (without defects) with up to 10,000 oscillators in simulations. The synchronization domains were identified by a synchronization threshold of $\sigma_t = 0.85$ and a clustering analysis on the resulting data. This isolates the synchronized regions via domain walls of lower synchronization. Synchronization is found to be highly robust, with domain size scaling approximately linearly with system size (see Fig. S7b). In particular, for systems with up to 100 particles, complete synchronization is observed, whereas for

$N > 1000$, the domain size scales approximately as $N_{domain} \simeq 0.5N$. In the intermediate range, $100 < N < 1000$, the system occasionally achieves full synchronization, but may also form smaller synchronized domains. Since the experimental setup lies within this range, large synchronized regions ($N_{\mathrm{domain}} \propto N$) are likely to emerge; however, the presence of unresponsive particles makes the formation of a fully synchronized cluster unlikely.

5. Squirmer Parameter β

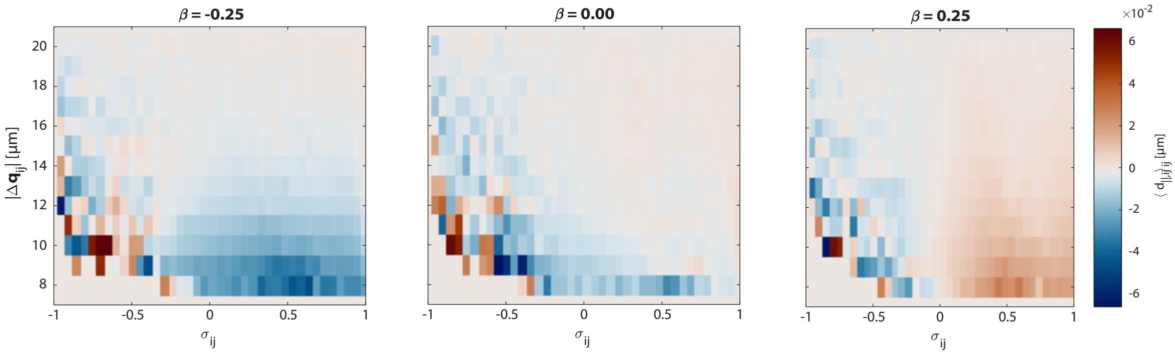

Fig. S9: Heat map of the mean parallel displacement $d_{\parallel,ij}$ for different synchronizations and distance of Delaunay neighbors in simulations, for squirmer parameter $\beta = [-0.25, 0.0, 0.25]$. For $\beta \leq 0$ (pusher), there is no region of net attraction, i.e. the swarmalators are always repelled. Source data are provided as a Source Data file.

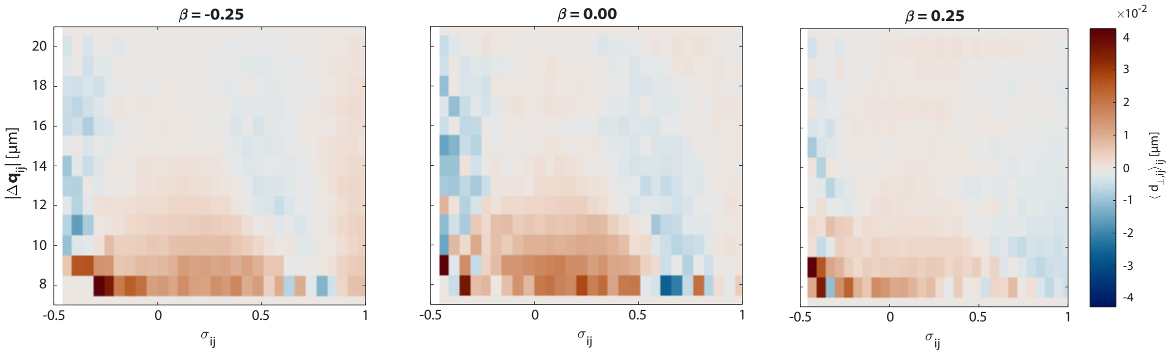

Fig. S10: Heat map of the mean perpendicular displacement $d_{\perp,ij}$ for different synchronizations and distance of Delaunay neighbors in simulations, for squirmer parameter $\beta = [-0.25, 0.0, 0.25]$. For $\beta > 0$ (puller), the data shows qualitative agreement with the experimental data (Fig. 5e in main text). Source data are provided as a Source Data file.

To understand the influence of hydrodynamic forces and synchronization on the overall motion of the particle around the reference point, we analyze the average displacement of the average particle position around the reference position as a function of synchronization and the neighbour distance, as shown in Fig. 4 of the main text. Here for each particle, we compute the displacement and synchronization averaged over 10 frames, and $\Gamma = 0$, so that the reference positions are fixed. Experimental data show that a pair of swarmalators is attracted when their average synchronization is zero, whereas increasing synchronization causes them to repel each other, resulting in synchronization-dependent pair coupling.

When this analysis is performed in simulations with $\beta = 0$, we do not observe any repulsive regime for the swarmalator pair; the pair is always (weakly) attracted for all values of synchronization, as shown in Fig. S9. To explore the possibility of a non-zero β, we perform simulations for $\beta \in (-1, 1)$. Fig. S9 shows that for pushers, with $\beta < 0$, the swarmalator pair remains attracted across all synchronization values. However, for pullers, with $\beta > 0$, a region of 'repulsive' coupling emerges at higher synchronization, consistent with the experimental observations.

This indicates that swarmalator interactions are highly sensitive to the hydrodynamic flow field — such that even a small change in β can have a non-trivial impact on the collective behavior of the swarmalators. Since the flow field of the light-driven colloids used in the experiment is expected to be similar to that of a neutral squirmer, we choose a small value of $\beta = 0.25$ to qualitatively reproduce the observed behavior. Note that a small positive squirmer parameter is consistent with the presence of a demixing bubble beneath the particle, which moves along with the ABP [6]. This generates an asymmetric flow field, with some qualitative resemblance to a (weak) puller.

To investigate the effect of hydrodynamic interactions on the motion of neighboring swarmalators — and, consequently, on the motility of the reference points — we consider a simplified model involving two fully synchronized swarmalators rotating around fixed reference positions. We assume that the reference points are fixed at $\mathbf{q}_1 = (0,0)$ and $\mathbf{q}_2 = (R,0)$, with the two swarmalators performing a circular trajectory around their respective reference points. With this, the orientation vectors are given by $\mathbf{e}_1 = \mathbf{e}_2 = \text{sign}(\omega)(\cos\omega t, \sin\omega t)$, and the difference vector $\hat{\mathbf{r}}(t) = \mathbf{r}_2(t) - \mathbf{r}_1(t)/|\mathbf{r}_2(t) - \mathbf{r}_1(t)| = \hat{x}$. The force on particle 2 due to particle 1 is then given by

$$\gamma^{-1}\mathbf{F}_{2\to 1} = \mathbf{U}_{flow,2}(r) = -\frac{p}{r^2}[1 - 3(\mathbf{e}_j \cdot \hat{\mathbf{r}})^2]\hat{\mathbf{r}} - \frac{s}{r^3}[\mathbf{e} - 3(\mathbf{e}_j \cdot \hat{\mathbf{r}})\hat{\mathbf{r}}], \tag{6}$$

where $\mathbf{U}_{lub,2}(r) = 0$ is assumed as they are fully synchronized. This then gives the time dependent force

$$\begin{aligned}\gamma^{-1}\mathbf{F}_{2\to 1} = &-\frac{p}{r^2}\left[1 - 3\cos^2\omega t\right]\hat{x} \\ &- \frac{s(\text{sign}(\omega))}{r^3}\left[\cos\omega t\hat{x} + \sin\omega t\hat{y} - 3\cos\omega t\hat{x}\right]\end{aligned} \tag{7}$$

Averaging this force over one cycle of rotation results in

$$\gamma^{-1}\mathbf{F}_{2\to 1} = \frac{p}{2r^2}\hat{x} = -\gamma^{-1}\mathbf{F}_{1\to 2} \tag{8}$$

This implies that for $p = 0$ (neutral squirmer), there is no net interaction between the rotating particles, i.e., $p \neq 0$ is necessary for the swarmalators to interact with each other (in the fully synchronized state). In particular, we find that for $p < 0$, i.e., $\beta > 0$ (puller), the swarmalator pair is attracted towards each other, thus rationalizing the experimental results observed and our choice of β in the simulation.

Simulation data for $\langle d_{\perp,ij}\rangle_{ij}$ shows that the lateral force also depends on β, with $\beta > 0$ and $\beta < 0$ resulting in opposite values for the perpendicular force component (Fig. S10). Again, the data for $\beta > 0$ shows a qualitative match with the experimental data. We can qualitatively understand this by assuming in our previous 'toy' model of two perfectly synchronized particles on circular trajectories, the center of rotation of the second particle is slightly displaced from the reference position. This implies that it performs a rotation around the point (R_0, ϵ), which would then give us a force

$$\gamma^{-1}\mathbf{F}_{2\to 1} = \frac{p}{2r^2}\frac{\hat{x} + \epsilon\hat{y}}{1 + \epsilon^2} = -\gamma^{-1}\mathbf{F}_{1\to 2}, \tag{9}$$

i.e., the perpendicular force (projected onto $\hat{y}$ – the connection vector perpendicular to $\mathbf{q}_1 - \mathbf{q}_2$) depends on the squirmer parameter p, thus rationalizing the data seen for $\langle s\rangle \simeq 1$ in Fig. S10. However, note that this simple model does not clarify where the angular velocity comes into play or how the perpendicular force is sustained (i.e., why it doesn't average out to zero).

6. Swarmalator motility in switching experiments

In the experiment that produced a state loop (see Fig. 5 in the main text), we switched Γ between 0.2 and -0.05. We selected these two values of Γ because they result in the same swarmalator motility and therefore let the Γ-switch only affect the attraction / repulsion behavior.

We use the mean squared displacement (MSD) to quantify the motility of the swarmalators. The MSD is defined as

$$\text{MSD}(t) = \langle(\mathbf{r}(t+\tau)_i - \mathbf{r}(\tau)_i)^2\rangle_{\tau,i} = \langle(x(t+\tau)_i - x(\tau)_i)^2 + (y(t+\tau)_i - y(\tau)_i)^2\rangle_{\tau,i} \tag{10}$$

where the data is averaged over time stamps τ and over all particles i. Fig. S11 shows the MSDs of swarmalators at the same density as used in the state-loop experiments for these two values of Γ.

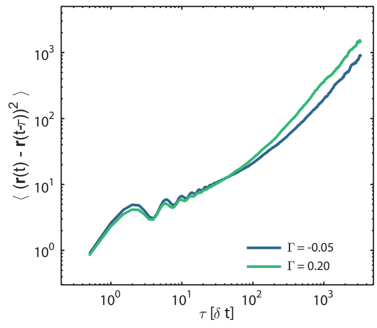

Fig. S11: Mean squared displacement (MSD) of the swarmalators at high density for $\Gamma = 0.2$ and $\Gamma = -0.05$. Source data are provided as a Source Data file.

7. Motivation of phenomenological swarmalator model

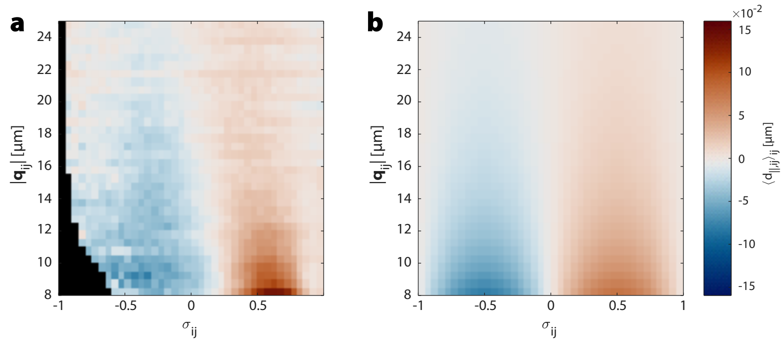

Fig. S12: **a** Heat map of the parallel displacement $d_{\parallel,ij}$ as a function of the synchronization σ_{ij} and reference point distance $|\mathbf{q}_{ij}|$ (as in Fig. 3e) **b** Fit to $d_{\parallel,ij}$ as a function of σ_{ij} and $|\mathbf{q}_{ij}|$ using the phenomenological equation $d_{\parallel,ij} = A \cdot \sin(\sigma_{ij}) \,/\, |\mathbf{q}_{ij}|^B$. Source data are provided as a Source Data file.

To obtain a simplified swarmalator model for our system, we need to formulate equations of motion for the positions $\mathbf{q}_i$ and phases θ_i of the swarmalators in terms of their synchronization and distance. Due to equation (2), the motion of swarmalator i is proportional to the displacement $\mathbf{d}_i$ of its mean ABP position $\mathbf{R}_i$ from its reference point $\mathbf{d}_i = \mathbf{R}_i - \mathbf{q}_i$ and we can write

$$\dot{\mathbf{q}}_i(t) = C\mathbf{d}_i(t) + \mathbf{F}_{rep,ij}(t) + \boldsymbol{\xi}_{i,q}(t). \tag{11}$$

Here, C is a scalar constant, $\mathbf{F}_{rep,ij}(t)$ is a short-ranged repulsive force between swarmalators and $\boldsymbol{\xi}_{i,q}(t)$ is Gaussian, δ-correlated noise, respectively. As explained in section 2.3, we found that $d_{\parallel,ij}$, the displacement $\mathbf{d}_i$ of swarmalator i projected on the direction of another swarmalators j, results from the hydrodynamic interactions of i with j and depends on their synchronization σ_{ij} and distance $\mathbf{q}_{ij}$. For a phenomenological model, we assume that the displacement $\mathbf{d}_i$ of a swarmalator i, which interacts with multiple neighbors N_j can be obtained by combining the displacements $d_{\parallel,ij}$ that result from the interaction with each neighbor:

$$\mathbf{d}_i(t) = \frac{1}{N_j} \sum_{j \neq i} d_{\parallel,ij}(t) \cdot \hat{\mathbf{q}_{ij}}(t). \tag{12}$$

Here, $\hat{\mathbf{q}_{ij}}$ is the direction from the reference point $\mathbf{q}_i$ of i to the reference points $\mathbf{q}_j$ of its neighbors j. We can therefore write the equation of motion for the position of i as

$$\dot{\mathbf{q}}_i(t) = \frac{C}{N_j} \sum_{j \neq i} d_{\parallel,ij}(t) \cdot \hat{\mathbf{q}_{ij}}(t) + \mathbf{F}_{rep,ij}(t) + \boldsymbol{\xi}_{i,q}(t). \tag{13}$$

This expression still relies on the displacement $d_{\parallel,ij}$. Figure S12a shows the mean measured parallel displacement $d_{\parallel,ij}$ of swarmalators (same as Fig. 3e in the main text). To obtain an expression for $d_{\parallel,ij}$

as a function of σ_{ij} and $\mathbf{q}_{ij}$, we fitted these values with the phenomenological equation

$$d_{\parallel,ij}(t) = A \cdot \sin\left[\sigma_{ij}(t)\right] \frac{1}{|\mathbf{q}_{ij}(t)|^{B}}, \tag{14}$$

where A and B are fitting parameters. The resulting fit is shown in Fig. S12 and yielded $A \approx 6.5$ and $B \approx 2$. Using this result together with the instantaneous synchronization $\sigma_{ij} = \cos(\theta_i - \theta_j)$, $\hat{q_{ij}} = \mathbf{q}_{ij}/|\mathbf{q}_{ij}|$ and $J = C \cdot A$ in equation (13), we obtain

$$\dot{\mathbf{q}}_i(t) = \frac{1}{N_j} \sum_{j \neq i} \frac{J\mathbf{q}_{ij}(t)}{|\mathbf{q}_{ij}(t)|^3} \sin\left[\pi \cos\left(\Delta\theta_{ij}(t)\right)\right] + \mathbf{F}_{rep,ij}(t) + \boldsymbol{\xi}_{i,q}(t), \tag{15}$$

which we use in equation (4) in the main text for our phenomenological swarmalator model, whose results are shown in Fig. 4 and Fig. S4. For a motivation of the equation of motion of the phases θ_i (equation (3) in the main text) we refer to SI section 4.

8. Swarmalator model and phase diagram

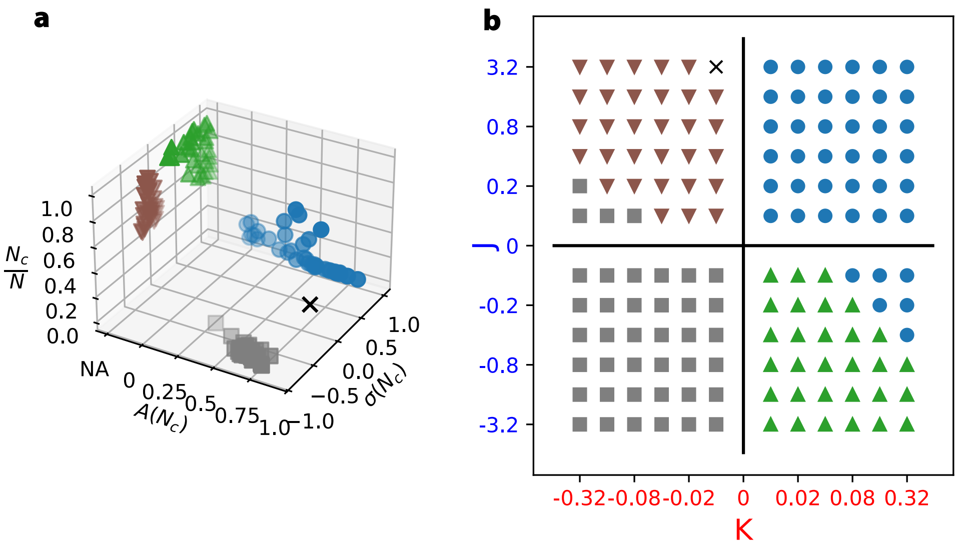

Fig. S13: **a** Phase diagram in the order-parameter space (see text), showing four well-separated phases. **b** Mapping of the phases from (**a**) onto the J-K parameter space, demonstrating that the four quadrants correspond directly to the four distinct phases – synchronized clusters (blue circles), dispersed–synchronized (green triangles), chain-like anti-synchronized (grey squares), and dispersed anti-synchronized states (brown inverted triangles).

To identify the different phases in the parameter space shown in Fig. 4 and Fig. S4, we perform a cluster analysis (on the last frame at time $20000/\omega_0$) for each parameter set and define the following order parameters:

- Average (weighted) cluster size N_c
- Average (weighted) asphericity $A(N_c)$
- Average (weighted) synchronization $\sigma(N_c)$

All data are weighted by the number of particles in each cluster. For example, for a cluster with asphericity A_i, a weight nc_i – the number of particles contained in the cluster – is assigned. A cutoff criterion is imposed to distinguish between clustered and non-clustered regimes. If more than half of the particles are isolated (i.e., not belonging to any cluster), the system is assumed to be in the dispersed phase. In this case, the asphericity is set to NA, the cluster size is re-defined as the number of isolated particles, and synchronization is computed locally within neighborhoods with a cutoff of 5σ, where σ is the particle size. When the majority of particles form clusters, the asphericity and synchronization

is calculated as a weighted average over all clusters. The cutoff used for the cluster analysis is 2σ. When the conformation data for each simulation is characterized by these order parameters, a clear separation of the data into four regions is observed, corresponding to four distinct swarmalator phases (see Fig. S13). We observe that for large values of $|K|$ and small J, the clustering phase extends into the dispersed phase. In this parameter region, the reduced motility of the particles slows down the dissolution of clusters, leading to long-lived clustered states even within dispersed regime. Notably, the chain-like anti-synchronized phase corresponds to clusters with high asphericity and smaller sizes, i.e., small and approximately linear arrangements of particles arising from geometric frustration. In contrast, the synchronized clusters span a broader range of asphericity values and typically form larger clusters.

9. Cluster rotation

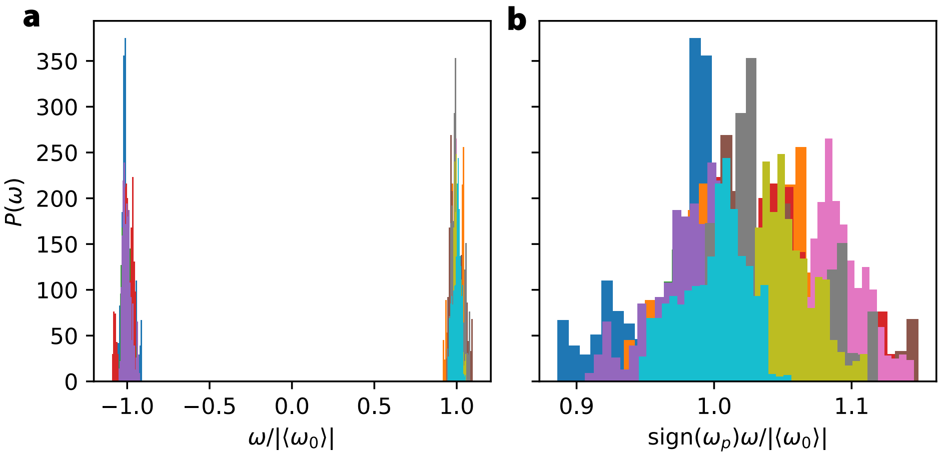

Fig. S14: **a** Probability distribution for the sign $\omega/|\langle\omega_0\rangle|$ of the angular velocity of the rotating cluster. **b** Probability distribution of the product $\mathrm{sign}(\omega_p)\,\omega/|\langle\omega_0\rangle|$, where ω_p and ω are the angular velocities of individual swarmalators and the cluster, respectively, showing a clear positive correlation. Different colors correspond to different realizations. Source data are provided as a Source Data file.

The emergent (long-time) rotation direction of a cluster can be either clockwise or counterclockwise, as shown in Fig. S14a. However, the cluster's rotation sense is directly correlated with the rotation sense of the individual swarmalators comprising the cluster, i.e., the sign of the angular velocity ω_p of an individual swarmalator. Examination of the histogram of the product $\omega\,\mathrm{sign}(\omega_p)$ reveals that all distributions collapse into the positive regime ($\omega > 0$), indicating that the cluster rotates in the same sense as its constituent swarmalators, see Fig. S14b. This is consistent with the heat map in Fig. 5e, which predicts that a synchronized pair with individual CW/ACW rotations rotates collectively in the same CW/ACW sense. It should be noted, however, that the individual swarmalators spontaneously adopt either clockwise or counterclockwise rotations, leading to clusters that are equally likely to rotate in either direction. This arises because the particles themselves are achiral; nevertheless, noise and hydrodynamic coupling allow the system to spontaneously order into a coherent left- or right-rotating states.

References

1. Golestanian, R., Yeomans, J. M. & Uchida, N. Hydrodynamic synchronization at low Reynolds number. *Soft Matter* **7,** 3074–3082 (Mar. 2011).
2. Niedermayer, T., Eckhardt, B. & Lenz, P. Synchronization, phase locking, and metachronal wave formation in ciliary chains. *Chaos: An Interdisciplinary Journal of Nonlinear Science* **18,** 037128 (Sept. 2008).
3. Maestro, A. *et al.* Control of synchronization in models of hydrodynamically coupled motile cilia. *Communications Physics* **1,** 28 (June 2018).
4. Guirao, B. & Joanny, J.-F. Spontaneous Creation of Macroscopic Flow and Metachronal Waves in an Array of Cilia. *Biophysical Journal* **92,** 1900–1917 (Mar. 2007).
5. Uchida, N. & Golestanian, R. Synchronization and Collective Dynamics in a Carpet of Microfluidic Rotors. *Physical Review Letters* **104,** 178103 (Apr. 2010).
6. Gomez-Solano, J. R. *et al.* Tuning the motility and directionality of self-propelled colloids. *Scientific Reports* **7,** 14891 (Nov. 2017).
