# Supplementary material for: Tunable colloidal swarmalators with hydrodynamic coupling
Source: Nat Commun. 2025 Dec 8;16:10984. doi: 10.1038/s41467-025-66830-5 (PMC12690114; doi:10.1038/s41467-025-66830-5)
Supplement: Supplementary file 2 — Description of Additional Supplementary Files [file 41467_2025_66830_MOESM2_ESM.pdf]

File Name: "Supplementary video 1 Hydrodynamic synchronization on a grid.mp4"
Description: **Hydrodynamic synchronization on a grid.** Shows the oscillation of ABPs around reference positions arranged on a hexagonal grid for a lattice spacing of $D = 12$, 18 and 24 µm in experiment and simulation. The ABPs are color coded according to their synchronization σ_i to their neighbors.
File Name: "Supplementary video 2 Interacting swarmalators with negative and positive coupling parameter Gamma.mp4"
Description: **Interacting swarmalators with negative and positive** Γ**.** Time evolution of exemplary runs during the first 8000 δt of dense systems of swarmalators for $\Gamma = -0.1$ and $\Gamma = 0.2$ in experiment and simulation (as in Fig. 3 in the main text). Swarmalators are colored according to their synchronization σ_i to their neighbors.
File Name: "Supplementary video 3 Animated phase diagram of the swarmalator model.mp4"
Description: **Animated phase diagram of the swarmalator model.** Shows the time evolution of dense swarmalator systems from numerical simulations of the swarmalator model given in equation (3) and equation (4) in the main text for the same values of K and J as in Fig. 4. The swarmalators are colored according to their phase.
